# Exploring the Diversity of the Human Blood Virome

**DOI:** 10.3390/v13112322

**Published:** 2021-11-21

**Authors:** María Cebriá-Mendoza, María A. Bracho, Cristina Arbona, Luís Larrea, Wladimiro Díaz, Rafael Sanjuán, José M. Cuevas

**Affiliations:** 1 Institute for Integrative Systems Biology (I2SysBio), Universitat de València-CSIC, 46980 València, Spain; maconce@alumni.uv.es (M.C.-M.); Wladimiro.Diaz@uv.es (W.D.); rafael.sanjuan@uv.es (R.S.); 2 Joint Research Unit “Infection and Public Health”, FISABIO-Universitat de València I2SysBio, 46020 València, Spain; alma.bracho@uv.es; 3 CIBER in Epidemiology and Public Health (CIBERESP), 46020 València, Spain; 4 Centro de Transfusión de la Comunidad Valenciana, 46020 València, Spain; arbona_cri@gva.es (C.A.); larrea_lui@gva.es (L.L.); 5 Department of Informatics, Universitat de València, 46020 València, Spain; 6 Department of Genetics, Universitat de València, 46020 València, Spain

**Keywords:** orphan virus, blood virome, anellovirus, pegivirus, virus discovery, metagenomics

## Abstract

Metagenomics is greatly improving our ability to discover new viruses, as well as their possible associations with disease. However, metagenomics has also changed our understanding of viruses in general. The vast expansion of currently known viral diversity has revealed a large fraction of non-pathogenic viruses, and offers a new perspective in which viruses function as important components of many ecosystems. In this vein, studies of the human blood virome are often motivated by the search for new viral diseases, especially those associated with blood transfusions. However, these studies have revealed the common presence of apparently non-pathogenic viruses in blood, particularly human anelloviruses and, to a lower extent, human pegiviruses (HPgV). To shed light on the diversity of the human blood virome, we subjected pooled plasma samples from 587 healthy donors in Spain to a viral enrichment protocol, followed by massive parallel sequencing. This showed that anelloviruses were clearly the major component of the blood virome and showed remarkable diversity. In total, we assembled 332 complete or near-complete anellovirus genomes, 50 of which could be considered new species. HPgV was much less frequent, but we, nevertheless, recovered 17 different isolates that we subsequently used for characterizing the diversity of this virus. In-depth investigation of the human blood virome should help to elucidate the ecology of these viruses, and to unveil potentially associated diseases.

## 1. Introduction

Viruses are ubiquitous in all natural environments and can be considered the major source of nucleic acids on earth [1]. On one hand, metagenomics has become an essential tool for pathogen discovery, potentially enabling a faster response to future outbreaks of infectious diseases in humans [2]. On the other hand, metagenomics has transformed our understanding of viral diversity [3], thus questioning the classical assumption of viral agents as pathogens [4]. Indeed, a new paradigm has emerged according to which viruses are integral components of ecosystems, sporadically causing diseases, but also providing beneficial effects to their hosts [5,6].

Metagenomics applied to human blood has revealed the presence of viral sequences from multiple families, including members of the Anelloviridae, Herpesviridae, Picornaviridae, Poxviridae, Flaviviridae, Marseilleviridae, Mimiviridae, and Phycodnaviridae families [7,8,9,10,11]. However, two distinct viral groups outstand as most abundant among chronic and/or asymptomatic infections in human blood. First, over 50% of the general population is infected with anelloviruses, although the reported prevalence varies greatly among populations [12]. Second, the prevalence of human pegivirus (HPgV), a flavivirus, ranges between 1 and 5% in healthy blood donors from developed countries but increases up to 20% in developing countries [13]. Since anelloviruses and HPgV are efficiently transmitted by the parenteral route [14,15], their prevalence is even higher among poly-transfused patients and intravenous drug users [16,17].

Human anelloviruses contain a circular single-stranded DNA genome ranging between 2.8 and 3.9 kb and can be found in most tissues, cells, and body fluids. Three different genera have been described so far within this family [12]: torque teno virus (TTV, Alphatorquevirus), torque teno mini virus (TTMV, Betatorquevirus), and torque teno midi virus (TTMDV, Gammatorquevirus). According to the International Committee on Taxonomy of Viruses (ICTV), TTV, TTMV, and TTMDV have been recently subdivided into 26, 38, and 15 species, although this diversity is expected to increase as new isolates are identified [18]. This recent classification is based on the analysis of the entire ORF1 nucleotide sequence using 69% pairwise sequence identity as a species demarcation criterion [19]. Human anelloviruses seem to be essentially innocuous [20], and some potentially beneficial effects have even been suggested [15], such as immune system maturation after newborn infection [20,21].

HPgV, also known as GB virus C, is the known human virus most closely related to hepatitis C virus [22]. HPgV is a lymphotropic virus with a 9.3 kb positive-sense ssRNA genome, organized similarly to hepatitis C virus, which is translated into a single polyprotein of approximately 3000 amino acids. Currently, HPgV has been subdivided into six genotypes showing different geographical distribution patterns and multiple subtypes [23,24]. As with anelloviruses, the World Health Organization (WHO) does not recommend blood screening for HPgV because it is not associated to any disease [16], apart from some weak evidence [14]. Indeed, HPgV seems to be protective against infection by human immunodeficiency virus [16], and co-infection in patients with Ebola virus disease has been associated with higher survival rates [25]. These pieces of evidence suggest that HPgV and human hosts may establish a mutually beneficial symbiotic relationship [16].

The analysis of human blood virome is of particular interest because potential transmission of unknown or unexpected viruses by blood transfusions or organ transplantations is a concern for public health systems [26]. In addition, these studies should improve our understanding of the mutualistic/commensal interactions between viruses and hosts [4]. To shed some light on this issue, we have recently implemented a protocol for viral enrichment using human plasma samples, which allows efficient recovery of DNA and RNA viruses [18]. Here, we have used this methodology to characterize blood virome diversity in a cohort of 587 pooled-plasma samples from healthy donors.

## 2. Materials and Methods

### 2.1. Sample Collection

A total of 587 plasma samples from healthy donors were collected from the Centro de Transfusión de la Comunidad Valenciana (Valencia, Spain) from 15 September 2018 to 30 March 2019 and stored at −80 °C until use. In accordance with the Declaration of Helsinki, all subjects provided written informed consent. The protocol was approved by the University of Valencia ethics committee (IRB No. H1489496487993). Plasma samples were divided into 60 pools, each containing between 8 and 13 samples (Supplementary Table S1).

### 2.2. DNA/RNA Extraction and Amplification

Each of the 60 pools (SP1-SP60) analyzed in this study was obtained by mixing 1 mL of plasma from a variable number of donors (between 8- and 13-mL total). To assess viral recovery, each pool was spiked with 10^3^ PFU of ϕX174 and 10^4^ PFU of vesicular stomatitis virus (VSV). The purification protocol has been previously described in detail [18]. Briefly, plasma pools were processed with 1.0 µM filters to remove cells and other non-viral particles and the filtered fractions were subject to high-speed centrifugation (87,000 *g*, 2 h, 4 °C), washed with PBS 1X (87,000 *g*, 1 h, 4 °C), and resuspended in 245 µL 1X digestion buffer (Turbo DNA Free kit, Ambion, Carlsbad, CA, USA). Then, 5 µL of Turbo DNase, 2 µL of Benzonase (Sigma, Darmstadt, Germany) and 2 µL of micrococcal nuclease (NEB) were added to the sample to remove unprotected nucleic acids. After incubation (1 h, 37 °C), 20 µL of stop reagent was added, following the manufacturer’s instructions. Then, 240 µL supernatant was transferred to a new tube and split into two fractions: 200 µL fraction was used for RNA extraction using TRIzol LS reagent (Invitrogen, Carlsbad, USA), followed by purification with the QIAamp Viral RNA Mini kit (Qiagen, Hilden, Germany) and amplification with the QuantiTect Whole Transcriptome kit (Qiagen), and 40 µL fraction was used for DNA extraction with the QIAamp Viral RNA Mini kit and amplification with the TruePrime WGA kit (Sygnis, Heidelberg, Germany). To control for environmental contaminants in materials and reagents, eight blank samples containing 10 mL PBS 1X were processed in parallel with the rest of the samples. Then, taxonomical information obtained from blanks was bioinformatically subtracted from actual samples.

### 2.3. Massive Parallel Sequencing

For each pool, DNA and RNA amplification products were mixed in equimolar concentration before library preparation, which was carried out using Nextera XT DNA library preparation kit with 15 amplification cycles (Illumina, San Diego, USA), and subject to pair-end sequencing in a NextSeq device. The raw sequence reads were deposited in the Short Read Archive of GenBank under accession number PRJNA731624.

### 2.4. Sequence Analysis

Sequence data were quality-checked using FastQC v0.11.9 (http://www.bioinformatics.babraham.ac.uk/projects/fastqc/, accessed on 20 November 2021) and MultiQC v1.8 [27]. Reads were quality-filtered using bbduk.sh from BBTools suite v38.82 [28]. A quality trimming threshold of 20 was used, and reads below 70 nucleotides in length were removed from the dataset.

Sequence identification was carried out using the Centrifuge software package [29] version 1.0.4 using a minimum exact match of 18. A customized database was generated from the NCBI nt database downloaded in September 2020. The Centrifuge download tool was used for incorporating archaea, viruses, bacteria, and fungi genomes from the September 2020 RefSeq database at the “Complete Genome” and “Chromosome” assembly levels. Centrifuge results were post-processed for contaminant removal and analyzed with Recentrifuge [30] version 1.3.2 using a minscore of 22.

Assembly was individually performed for each pool with metaSPAdes [31] version 3.15.0 using default parameters. Homology analysis of the contigs was performed against a local copy of the NCBI nucleotide (nt) database using BLASTn v2.10.0 with an E-value cutoff of 10^−5^. Average coverage depth was estimated using bbmap.sh from BBTools suite v38.68. The newly described sequences belonging to anelloviruses, HPgV, and a single microvirus were deposited in GenBank under accession numbers MZ285962-MZ286225 (Supplementary Table S2), MZ420565-MZ420581 (Supplementary Table S3), and MZ286294, respectively.

Putative open reading frames were identified using ORF Finder (https://www.ncbi.nlm.nih.gov/orffinder/, accessed on 20 November 2021).

### 2.5. Phylogenetic Analysis

To study phylogenetic relationships within the family Anelloviridae, nucleotide ORF1 sequences from hominid TTV, TTMV, and TTMDV accepted as reference species by ICTV were downloaded (Supplementary Table S4). Regarding HPgV phylogenetic analysis, nucleotide sequences of the complete polyprotein corresponding to isolates available from Genbank by March 2021 were downloaded (Supplementary Table S3). Sequence alignment (based on the amino acid sequences) was performed with MUSCLE [32] as implemented in MEGA version X [33], and subsequent phylogenetic inference using nucleotide sequences was conducted with the maximum likelihood (ML) method, also implemented in MEGA version X. Analyses were performed using the best-fit nucleotide substitution model, identified as GTR+Г+I using Akaike information criterion. The reliability of the phylogenetic results was assessed using 1000 bootstrap pseudo-replicates. The final trees were annotated with EvolView [34]. Anellovirus species demarcation was performed by checking nucleotide pairwise identity matrices obtained independently for each genus.

### 2.6. Sanger Sequencing

For HPgV, missing regions from the polyprotein sequence were read by Sanger sequencing using specific primers designed based on known sequence regions. First, the RNA sample from each HPgV positive pool was subject to reverse transcription using Superscript IV (Invitrogen, Carlsbad, USA) and random hexamers, following manufacturer instructions. Then, for each missing region, 25 µL PCR reactions were performed adding 2 µL of cDNA product, Phusion High-fidelity DNA polymerase (ThermoFisher Scientific, Vilnius, Lithuania), and GC buffer using specific annealing conditions for each amplification product. PCR primers were used for Sanger sequencing. For SP30, SP49, and SP53 pools, individual detection of HPgV positive samples from each pool was done using specific primers (Forward 5′-CAGAACCATACAGCCTATTGTGA-3′ and Reverse 5′-CACCTTAGATCCCCAGCCCA-3′) designed from conserved regions in the global alignment used to obtain Figure 5.

### 2.7. Split Network Analysis

A phylogenetic network for HPgV was estimated using SplitsTree4 program (version 4.17.0) [35] based on the HPgV sequence alignment used for the ML phylogeny. NeighborNet method was used to calculate the reticulate phylogeny. The GTR+Г+I model was applied with parameters Г=0.7028 and I=0.5234.

## 3. Results

### 3.1. Overall Sequence Output

We used a recently described [18] experimental protocol for viral fraction enrichment. Briefly, 60 plasma pools from 8–13 individual plasma samples obtained from healthy donors were analyzed. Pools were filtered to remove bacteria and cellular debris, subjected to high-speed centrifugation to pellet potential viruses and treated with nucleases to digest free nucleic acids, followed by extraction of both viral DNA and RNA independently. Finally, the extracted nucleic acids were subjected to random ϕ29-based amplification [36] and library preparation. To monitor the efficiency of virus recovery, all plasma pools were initially spiked with 10^3^ PFU/mL of bacteriophage ϕX174 (non-enveloped, circular single-stranded DNA virus) and 10^4^ PFU/mL of vesicular stomatitis virus (VSV, enveloped, linear single-stranded RNA virus). Since the purification protocol could carry over residual amounts of non-viral nucleic acids, eight blank controls were processed in parallel to evaluate contamination risk. The reads obtained in these controls were used for taxonomic classification and computational subtraction of these potential contaminants.

**Figure 1:**
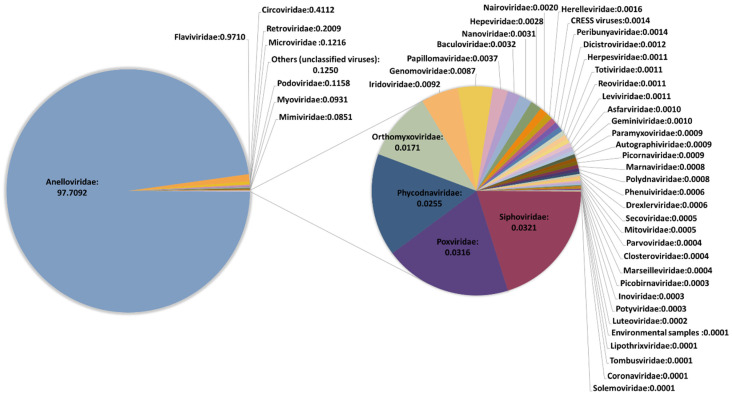
Description of the virome characterized in this study. The classification is shown at the family level. Frequencies were obtained excluding spiked viruses.

We performed an initial taxonomic classification using Centrifuge software [29] to select viruses, and then removed potential contaminations using Recentrifuge [30]. The ϕX174 and VSV reads were used for assessing the recovery efficiency of DNA and RNA viruses, respectively. Reads classified as belonging to Anelloviridae family were detected in all but one pool (Table 1 and Supplementary Tables S4 and S5), with read numbers ranging from 10 to 1,580,534. No clear conclusions could be drawn when checking the presence of the spiked DNA virus ϕX174, since it was present in the pool showing no anellovirus reads but absent in four pools in which anellovirus reads were detected (Supplementary Tables S4 and S5). In turn, HPgV reads were detected in 17 pools, one of them showing only 9 reads and the rest ranging between 339 and 25,965 reads. When checking the spiked RNA virus VSV, no significant differences were observed in the number of reads between pools detecting HPgV and the rest of the pools (*t*-test: *p* = 0.37), which suggested that HPgV detection was not subject to significant experimental bias. Other viruses were detected in all pools but represented a very low fraction. In fact, when globally considering the results of our study, 97.71% of viral reads corresponded to anelloviruses, 0.97% belonged to HPgV, and the remaining 1.32% included 46 viral families (Figure 1 and Supplementary Table S4). The remarkable diversity of this residual fraction strongly suggested that these reads may correspond to taxonomic misidentifications or amplification of small traces of nucleic acids present in the reagents used in our virus-enrichment protocol and that were not efficiently removed computationally. This was supported by the fact that ambiguities in the taxonomical classification of reads were not properly handled by Recentrifuge [18], limiting our ability to remove potential contaminations corresponding to phylogenetically unclassified reads. A clear example of this is the detection of Circoviridae family, which represented the third most abundant family in our study, and which has been previously associated with contaminating reagents [37]. In addition, most of the identified taxonomical groups corresponded to viruses infecting bacteria, algae, protozoa, and fungi. For those reads potentially associated with human pathogens, mapping to the corresponding reference sequences assigned by Centrifuge was unsuccessful, indicating errors in taxonomic classification.

Interestingly, a full genome from a circular single-stranded DNA bacteriophage belonging to the family Microviridae was recovered from two pools (SP47 and SP57), with >1000 reads belonging to this virus in each of the pools. A blastp analysis of the six putative ORFs showing homology with microvirus sequences from databases yielded identities ranging between 48.3 and 61.5% with the closest reference sequence (Supplementary Table S6). This result highlights the sensitivity of our purification protocol, which was able to recover complete genomes from viruses that are likely to stem from contamination. Alternatively, this microvirus might be a true component of human blood.

Finally, although our experimental approach allowed the detection of large viruses [18], only marginal evidence of the presence of giant blood Marseille-like viruses was obtained (Supplementary Table S4), in agreement with previous studies [38,39] suggesting that this signature could also be a laboratory contaminant.

### 3.2. Phylogenetic Analysis of Anelloviruses

For each of the 60 pools, we generated contigs from all reads regardless of their preliminary taxonomical classification, which is an effective approach for the detection of new anelloviruses [18,40]. Blast analyses allowed the detection of spiked and HPgV viruses, but most contigs corresponded to anelloviruses. Specifically, 332 contigs were assigned to this family, of which 69 showed overlapping ends and could, thus, be considered as complete genomes (Supplementary Tables S5 and S2). A significantly positive correlation was observed between the number of contigs and the total amount of anelloviral reads in each pool (Spearman’s correlation: *ρ* = 0.414; *p* = 0.001). The full-length ORF1 was obtained for 315 of the 332 contigs (94.9%). These were subsequently used for phylogenetic analysis and identification of new species. Initially, we constructed a maximum likelihood (ML) phylogenetic tree, including the reference species recently proposed by ICTV (Supplementary Table S7), which allowed assignment of our contigs as belonging to TTV, TTMV, or TTMDV genera (160, 111, and 61 sequences, respectively; Supplementary Table S2 and Supplementary Figure S1). Sixty-seven of the 69 contigs considered as complete genomes belonged to TTMV genus, and a single contig was assigned to each TTV and TTMDV genera. This is consistent with the presence of shorter GC-rich regions in TTMV [41], which can increase assembly efficiency, as previously described [18].

The methodology established for anellovirus species classification has been modified recently and the number of reference species has been updated accordingly. Consequently, we decided to reevaluate the data of a recent study in which we applied the same viral enrichment experimental and bioinformatics procedures to a smaller number of samples [18]. This reevaluation yielded 26 new species (6, 11, and 9, for TTV, TTMV, and TTMDV, respectively; Table 2 and Supplementary Tables S8–S10), which were subsequently included in the pool of reference species used for characterizing the sequences analyzed in the present study. Additionally, a comparison between our previous and current results could shed some light on the level of anellovirus diversity which remains to be discovered in the local population that we analyzed.

**Figure 2:**
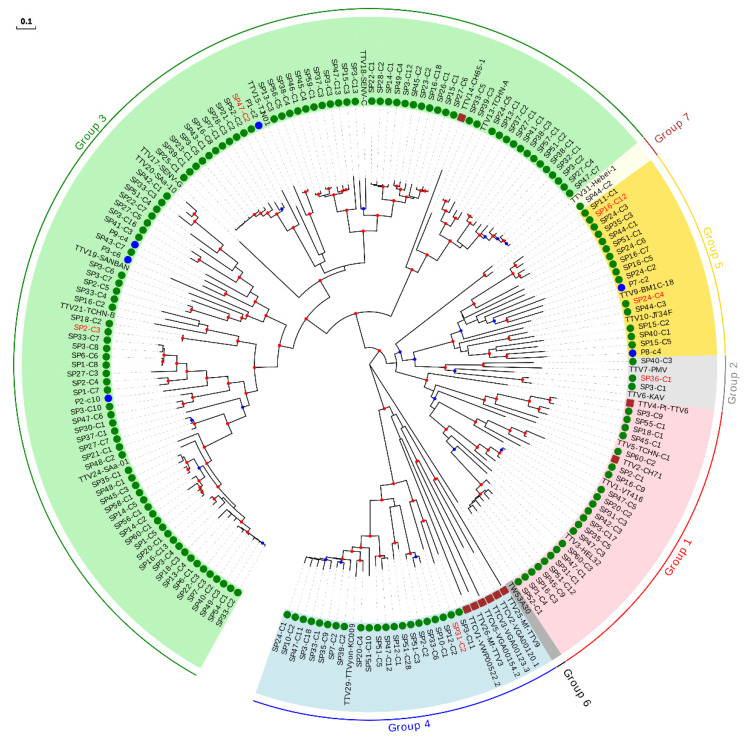
Phylogenetic tree of ORF1 sequences belonging to the TTV genus. Sequences described in this study are marked with a green circle. Those sequences that could be considered as new species are labeled in red. Sequences identified as new species after reevaluating data from our previous study [18] are marked with a blue circle. Non-hominid primate isolates are marked with a brown square. Nodes supported by bootstrap values ranging 0.7–0.85 and 0.85–1.0 are indicated with blue and red circles, respectively. The scale bar indicates the evolutionary distance in nucleotide substitutions per site.

For the sake of clarity, the characterization of our sequences was done by constructing independent ML phylogenetic trees and identity matrices for each genus, which only included isolates considered as reference species and those described after reevaluation of our previous study [18] (Supplementary Table S7). For the TTV genus, which is proposed to consist of seven phylogenetic groups [42], the tree included our 160 new sequences, 26 reference species, and the six newly described species (Figure 2, Table 2, and Supplementary Table S11). This tree, along with pairwise identities values, indicated that 23 of our sequences could be considered as belonging to six novel species (Table 2 and Supplementary Figure S2), whereas the remaining sequences clustered within 62.5% (20 out of 32) of the reference species, although this percentage increased up to 87.0% (20 out of 23) when excluding non-hominid primate isolates, which were not related with any of our sequences (Figure 2 and Supplementary Table S7). TTV variability obtained in our study covered a major fraction of the worldwide diversity for this genus, but the distribution of sequences within each species was highly variable (Supplementary Figure S2 and Supplementary Table S11). For instance, four species clustered with only one of our sequences whereas the species represented by isolates TTV24-SAa-01, TTV18-SENV-C, and TTV29-yon-KC009 clustered with 25, 20, and 18 of our sequences, respectively. Globally, our sequences clustered within species belonging to all proposed TTV groups, except for group 6, which only includes one isolate identified in eastern Taiwanese people [42] and that is not currently considered as a reference species by ICTV. Overall, we found a significantly positive correlation between the number of species included in each group and the number of newly described sequences (Spearman’s correlation coefficient; *ρ* = 0.971; *p* < 0.01).

**Figure 3:**
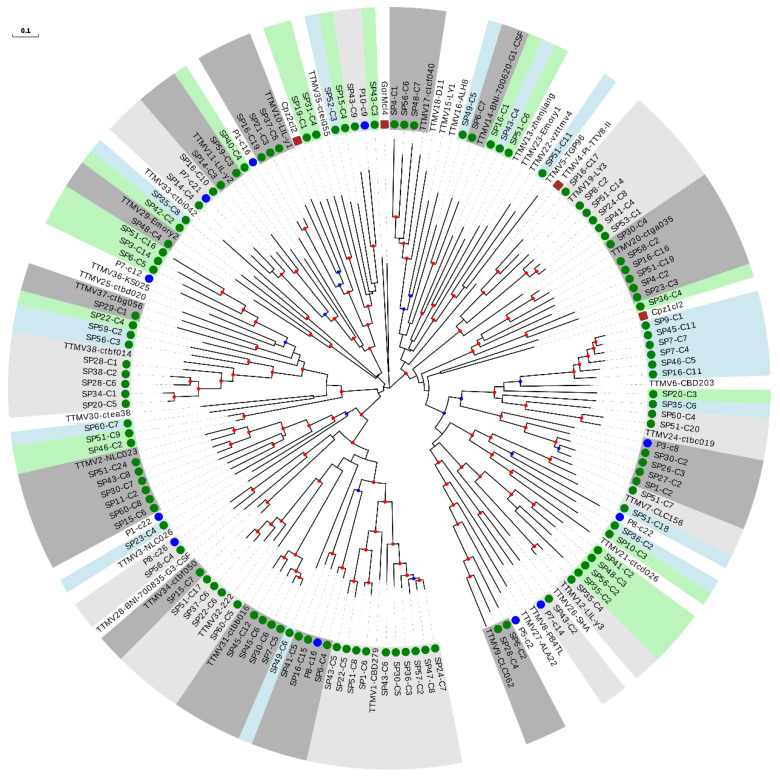
Phylogenetic tree of ORF1 sequences from the TTMV genus. Sequences described in this study are marked with a green circle. Sequences identified as new species after reevaluating data from our previous study [18] are marked with a blue circle. New species (including one or more new sequences) are indicated with background green or blue color in order to distinguish contiguous clusters. Clusters of representative species including new sequences are indicated with background light or dark grey colors in order to distinguish contiguous clusters. Non-hominid primate isolates are marked with a brown square. Nodes supported by bootstrap values ranging 0.7–0.85 and 0.85–1.0 are indicated with blue and red circles, respectively. The scale bar indicates the evolutionary distance in nucleotide substitutions per site.

We then constructed a phylogenetic tree with the 111 sequences from our study belonging to the TTMV genus, 38 reference species, and the 11 newly described species (Figure 3 and Table 2). Interestingly, 40 of our sequences could be considered as belonging to 27 novel species (Table 2 and Supplementary Table S12), which strongly increased the TTMV diversity described so far. The remaining 71 sequences clustered within 49.0% (24 out of 49) of the included species, and this percentage increased up to 53.3% (24 out of 45) when excluding non-hominid primate isolates (Figure 3 and Supplementary Table S7).

**Figure 4:**
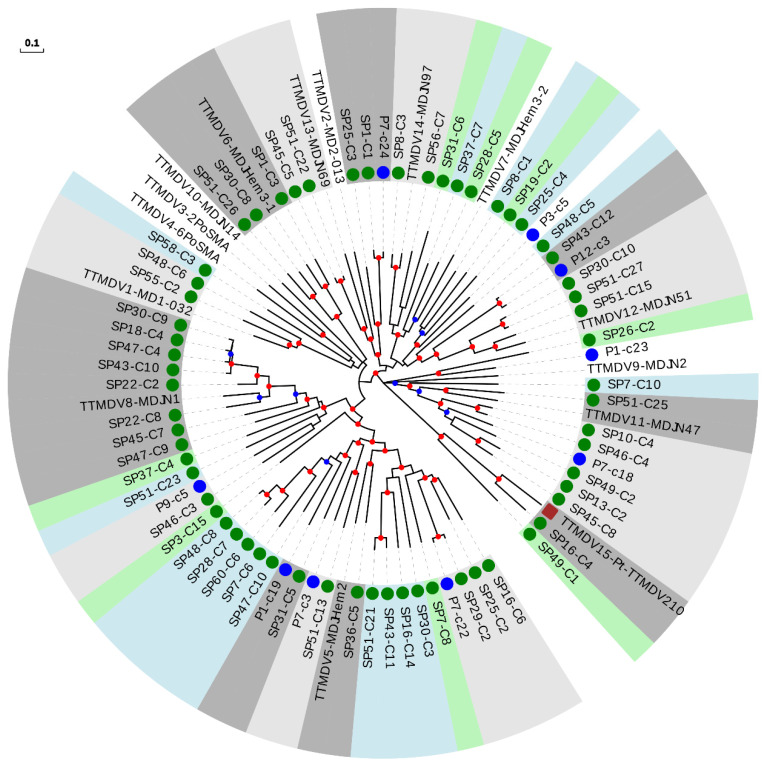
Phylogenetic tree of ORF1 sequences from the TTMDV genus. Sequences described in this study are marked with a green circle. Sequences identified as new species after reevaluating data from our previous study [18] are marked with a blue circle. New species (including one or more new sequences) are indicated with background green or blue color in order to distinguish contiguous clusters. Clusters of representative species including new sequences are indicated with background light or dark grey colors in order to distinguish contiguous clusters. The non-hominid primate isolate is marked with a brown square. Nodes supported by bootstrap values ranging 0.7–0.85 and 0.85–1.0 are indicated with blue and red circles, respectively. The scale bar indicates the evolutionary distance in nucleotide substitutions per site.

For the TTMDV genus, we constructed a tree including our 61 newly described sequences, 15 reference species, and the 9 newly described species (Figure 4 and Table 2). Twenty-four of our sequences could be assigned to 17 novel species (Table 2 and Supplementary Table S13), substantially increasing known TTMDV diversity, similar to what we observed for TTMV. The remaining 37 sequences clustered within 66.6% (16 out of 24) of the included species, surprisingly also including the only non-hominid primate isolate described for TTMDV.

The reevaluation of our recent study led to the identification of 26 potential novel species, most of them belonging to TTMV and TTMDV genera (Table 2). Despite the incorporation of these proposed new species into the pool of reference species, 50 novel species were still identified in our new set. Although nearly half of the sequences were assigned as TTV, only six of the 50 novel species described here corresponded to this genus. For TTV, the percentage of novel species described decreased from 8.8% in our previous study to 3.8% in this study, suggesting that a significant fraction of the actual diversity of this genus has been already described, at least in the local population under study. When doing this comparison for TTMV and TTMDV, the percentages of novel species were moderately higher in our previous study (37.9 and 52.9%, respectively) than in the current study (24.3 and 27.9%, respectively). These results strongly suggest that the actual variability of these two genera in human is still far from being described. TTMV and TTMDV show lower prevalence in the human population than TTV [43], complicating viral detection. Alternatively, their prevalence could be similar to that of TTV but with a lower average load in infected people, again complicating detection, particularly in studies that do not implement efficient viral enrichment protocols.

No evidence of geographical compartmentalization of the described sequences was observed (Supplementary Table S7). To test this, we constructed two-by-two contingency tables in which reference species were classified according to whether they clustered with any of our sequences and whether or not they had European origin. This revealed no significant associations (Fisher’s exact tests, *p* > 0.05 for all analyses performed globally and independently for each anellovirus genus). A clear piece of evidence of this lack of association is that the species clustering with a higher number of sequences for each genus (TTV24-SAa-01, TTMV1-CBD279, and TTMDV8-MDJN1, with 25, 10, and 8 sequences, respectively) were of Asian origin (Figure 2, Figure 3 and Figure 4 and Supplementary Table S7). Interestingly, we also found one TTV sequence which clustered with the recently proposed group 7 detected in Eastern Taiwan indigenes [42] (Figure 2).

PCR assays for differential detection of human anelloviruses have shown that TTV and TTMV DNA is present at high prevalence in chimpanzees [44], which suggests the occurrence of cross-species transmission. In agreement with this, phylogenetic analysis shows that both non-hominid TTVs and TTMVs are interspersed with human TTVs and TTMVs, respectively, although none of the sequences described in this study clustered within non-hominid isolates (Figure 2 and Figure 3). On the contrary, it has been proposed that chimpanzee and human TTMDV are separate [44], although this could be a consequence of poor sampling with respect to TTV and TTMV. In agreement with this second possibility, we detected a cluster including the only chimpanzee isolate and one of our TTMDV sequences (Figure 4 and Supplementary Table S13). This result strongly suggests that phylogenetic relationships between human and non-hominid isolates are similar for the three genera and that apparent differences are likely due to variations in sampling success.

### 3.3. Analysis of HPgV

Seventeen pools were positive for HPgV (Table 3). After excluding pool SP16, which only showed nine HPgV reads, the rest of positive pools presented genome coverages ranging between 70.2 and 99.6% of the complete reference genome (Accession U44402 was used as reference sequence) and average depth coverages ranging between 12.4X and 1010.7X (Table 3). For pool SP16, a single contig of 518 bases was obtained and subsequently identified as belonging to genotype 2 after blast analysis. For pool SP53, the consensus sequence obtained revealed the presence of 219 ambiguities, which could be caused by the simultaneous detection of two different HPgV isolates. To confirm this, RNA was individually extracted from the ten plasma samples included in this pool, cDNA was obtained and an HPgV specific PCR using conserved primers was performed. Two HPgV positive samples were identified in this pool, supporting our initial conclusion. We performed a contig analysis for this pool, which detected the presence of two different haplotypes partially covering HPgV genome. Then, specific PCRs and Sanger sequencing were done from individual cDNA samples to recover missing regions and unambiguously assign detected contigs.

**Figure 5:**
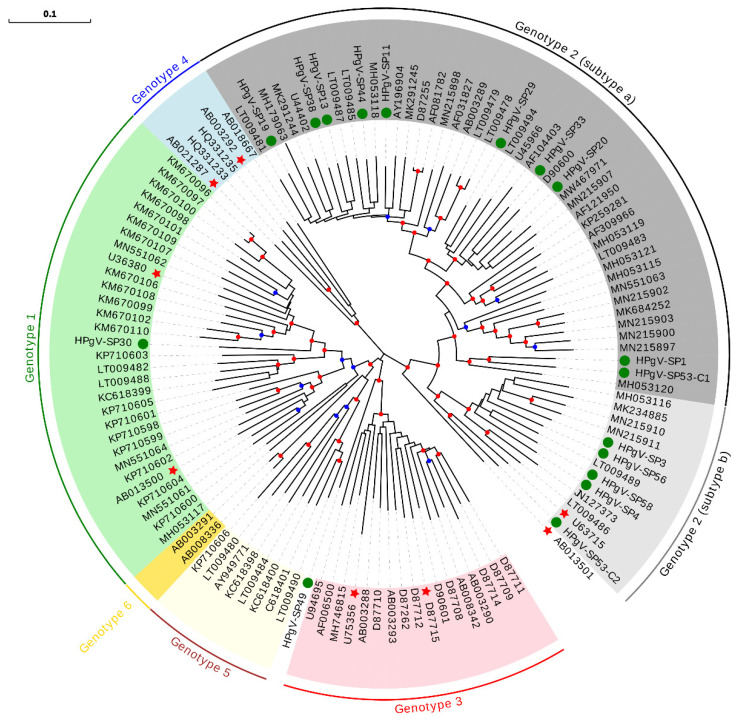
HPgV phylogeny of the polyprotein nucleotide sequence including all currently available isolates. Sequences described in this study are indicated with a green circle. Isolates previously reported as recombinants are indicated with a red star. For genotype 2, 2a and 2b subtypes are explicitly shown. Bootstrap values ranging 0.75–0.9 and 0.9–1.0 are indicated with blue and red circles, respectively. The scale bar indicates the evolutionary distance in nucleotide substitutions per site.

Overall, the HPgV prevalence was 3.1%, consistent with previously reported rates in a Spanish population [45]. Except for pool SP16, specific primers were designed using HPgV partial sequences from each pool for PCR amplification and Sanger sequencing of full-length coding genome sequences, which yielded 17 different isolates, two of them belonging to pool SP53. For ML phylogenetic analysis, nucleotide sequences of the complete polyprotein, which encompasses about 90% of the genome, were downloaded for all currently available isolates (Supplementary Table S3). This analysis showed that 15 of our sequences belonged to genotype 2 (Figure 5), with 10 and five sequences classified as subtypes a and b, respectively. HPgV-SP30 sequence was classified as belonging to genotype 1, and HPgV-SP49 sequence fell into a basal position relatively close to genotype 3.

The intermediate position of HPgV-SP30 and HPgV-SP49 among well-supported clusters in the ML phylogeny could point to recombinant sequences. We, thus, analyzed the treelikeness of the ML phylogeny (Figure 5). The phylogenetic network (Supplementary Figure S3) showed that both HPgV-SP30 and HPgV-SP49 seemed to be involved in a reticulate evolutionary history underlying recombinant events. Further recombination analysis (data not shown) performed with RDP4 software [46] suggested that HPgV-SP49 is an intergenotype recombinant (genotype 1/genotype 3), while HPgV-SP30 is an intra-genotype 1 recombinant. To discard that recombinant sequences detected in these two pools were actually caused by the presence of two different HPgV isolates in different samples from each pool, RNA was individually extracted from the ten plasma samples included in each pool, cDNA was obtained, and an HPgV-specific PCR using conserved primers was performed. Only one HPgV positive sample was identified in each pool, supporting our conclusions.

## 4. Discussion

Viral diversity is clearly underappreciated [47]. Before the advent of metagenomics, PCR using degenerate primers targeting conserved regions was the most efficient method for virus discovery [48]. However, this approach can introduce a strong sampling bias in markedly heterogeneous groups, such as anelloviruses [12]. Although biases still exist, such as preferential amplification of circular DNA viruses, viral metagenomics provides a more powerful tool for viral discovery, where many of the differences in detection rates result from natural parameters, such as viral load or particle stability.

Anelloviruses are an ancient family characterized by a vast diversity [49], and it is believed that their evolution has followed that of the animals they infect [50,51]. In primates, this coevolution hypothesis was questioned since human and chimpanzee isolates did not group phylogenetically according to host species [44], except in the case of TTMDV, whose divergence has been proposed following the speciation of humans and chimpanzees. However, our results clearly suggest a common origin, since these initially apparent discrepancies among the different primate anellovirus genera are likely due to sampling bias. In agreement with this, we have shown that TTMV and TTMDV, discovered three and ten years after TTV [12], respectively, are characterized by a remarkable diversity that was previously undetected due to amplification bias. At this point, it is also worth mentioning that lower TTV viral loads have been observed in human plasma relative to other body compartments [52], which is also likely to be the case with TTMV and TTMDV. This might even have resulted in an underestimation of the actual anellovirus diversity in our study.

Initially, it was proposed that TTV genotypes presented differences according to geographical distribution, but these studies mainly relied on specific primers and, thus, were subject to amplification bias [12]. Our results suggest that anellovirus diversity lacks geographical compartmentalization, at least in general terms. This is particularly remarkable for TTV, since 87% of worldwide described human species have been identified in our study. In addition, anellovirus prevalence is highly variable and non-sequence specific amplification methods are required to avoid strong bias. The high prevalence of anelloviruses is a consequence of the multiple transmission routes used by these viruses, including parenteral, sexual, and vertical routes, in combination with an extensive polytropism [52]. For TTV, available data on prevalence, tropism, and pathogenicity are highly contradictory, precluding an unambiguous assessment of the impact of TTV persistence on pathology in humans [52].

Since TTV viral loads increase in immunosuppressed patients, it has been suggested that pathogenesis may be conditional [52], acting as an aggravating factor or as an opportunistic agent [53]. In this sense, the extensive anellovirus diversity obtained in studies that have implemented viral fraction enrichment, as in the present study, could provide clues about potential associations between certain variants and pathologies. In any case, anelloviruses are commonly considered part of the natural human virome due to their high prevalence and largely asymptomatic persistence. Indeed, it has been proposed that TTV load could be used as an endogenous marker of immune status, which can be useful for public health purposes. For instance, the TTV DNA level in the blood of patients undergoing organ transplantation may be used to monitor the patient response to treatment [54,55].

Lack of pathogenicity is one of the defining criteria of pegiviruses [13], although the discovery of a horse pegivirus associated with acute hepatitis outbreaks [56] suggests that at least one member of the Pegivirus genus can be pathogenic. Recently, a second human pegivirus, HPgV-2, has been described in tight association with hepatitis C virus infection [11]. We have not detected this new virus in our study, since it presents a very low prevalence in the general population [57]. In any case, HPgV-2 is still considered a pathologically orphan virus. HPgV seems to be an ancient human virus, and its worldwide genotype distribution is concordant with ancient human migrations [58,59]. For instance, ancestral migrations between African and southeastern Asian areas could account for genotype 3 distribution [58]. HPgV infection may persist for decades, but most healthy individuals clear viremia within 2 years of infection [14]. The evaluation of molecular and/or serological HPgV prevalence has shown large variability in the general population [22]. The prevalence observed in our study is in agreement with results showing that viral RNA is unfrequently detected among healthy blood donors [60], and with previous prevalence values reported in Spanish populations [45].

The relatively low number of HPgV full-length coding sequences available in public databases shows a clear predominance of genotypes 2 and 3, probably as a result of increased sampling in geographical regions where these genotypes are more abundant. The predominance of genotype 2 isolates in our data is consistent with studies from other European countries [57,61]. This bias can confound certain analyses, such as the higher genetic diversity reported for genotype 1 [62]. Besides, the detection of recombination can be difficult among highly similar viral variants, as is the case of many HPgV sequences [24]. Despite these difficulties, it has been shown that recombination is probably responsible for phylogenetic incongruence among HPgV subgenomic regions, both at an intra- and inter-genotype levels [62,63,64,65,66,67]. Although it is clear that recombination has not been pervasive enough to obscure HPgV population structure [63], it is an important factor to be considered when defining new isolates. In this sense, several studies have suggested that HPgV genotype may impact HIV disease [68,69,70,71], but others have not found such potential association [72,73]. In addition, unofficial ICTV designations of some isolates (i.e., isolates with accession numbers U63715, AB021287, and AB003292) actually correspond to recombinant sequences [62,64]. Consequently, to clarify associations between HPgV genotypes and disease, it is necessary to perform accurate taxonomical classification using complete or nearly complete genomes, as well as to check for potential recombination effects. This is also important when considering the potential use of HPgV in vaccination strategies complementing anti-HIV therapy [74].

The potential symbiotic or commensal role of HPgV could be related to reduced immune activation [75,76]. However, this might also explain the observed association between HPgV infection and non-Hodgkin’s lymphoma [77,78]. Recent discoveries of new closely related pegiviruses in several species [79,80] raise the possibility of implementing animal infection models which could help elucidate the potential benefits of HPgV chronic infection.

## 5. Conclusions

The viruses described in the present study have shown that blood samples from the general population harbor a remarkable anellovirus diversity. Until recently, pathogenesis has been the main target of viral studies, but this traditional view is changing due to the increasing number of viruses in healthy individuals revealed by metagenomics. Consequently, a different framework that considers viruses as often innocuous or, more interestingly, as potentially beneficial agents deserves further investigation.

## Figures and Tables

**Table 1:** Summary of virome composition for the 60 pools analyzed. Read numbers are given. For comparison, the number of reads for the eight blank controls processed are also shown and subsequently used for computational subtraction of potential contaminants.

Pool	Anellovirus Reads	Pegivirus Reads	Other Viruses	Pool/Blank	Anellovirus Reads	Pegivirus Reads	Other Viruses
SP1	101,069	25,965	64	SP35	419,986	0	317
SP2	1,580,534	0	3013	SP36	185,281	0	144
SP3	131,969	3669	421	SP37	666,063	0	1311
SP4	9992	4250	61	SP38	242,853	2261	479
SP5	47,927	0	225	SP39	15,756	0	200
SP6	718,633	0	330	SP40	342,193	0	3390
SP7	63,139	0	80	SP41	169,614	0	2815
SP8	76,204	0	5089	SP42	4519	0	118
SP9	153,491	0	52	SP43	206,185	0	99
SP10	30,175	0	1649	SP44	7975	10,713	19
SP11	9787	5706	143	SP45	124,171	0	210
SP12	57,559	0	15,397	SP46	29,728	0	431
SP13	95,922	1173	4844	SP47	150,531	0	3731
SP14	271,731	0	1757	SP48	45,430	0	676
SP15	141,896	0	37	SP49	94,919	5226	255
SP16	149,985	9	2610	SP50	0	0	340
SP17	10	0	74	SP51	299,530	0	17
SP18	24,134	0	7168	SP52	59,852	0	16
SP19	74,391	339	9506	SP53	14,323	5344	68
SP20	73,067	373	21	SP54	2404	0	7634
SP21	124,389	0	4952	SP55	663	0	121
SP22	51,168	0	3428	SP56	25,673	3523	131
SP23	51,730	0	557	SP57	52,296	0	1737
SP24	71,389	0	7583	SP58	1232	2158	241
SP25	4269	0	262	SP59	157,753	0	46
SP26	27,676	0	84	SP60	36,470	0	324
SP27	7659	0	3030	C01	0	0	593
SP28	96,187	0	270	C02	0	0	8022
SP29	334,689	6606	18,366	C03	0	0	76,410
SP30	69,110	6924	156	C04	0	0	3589
SP31	332,437	0	816	C05	0	0	93,531
SP32	1011	0	223	C06	0	0	4588
SP33	72,784	2033	68	C07	0	0	2964
SP34	270,083	0	57	C09	0	0	6731

**Table 2:** Summary of anellovirus analysis. ^1^ Number of reference species currently accepted by ICTV for each genus. ^2^ Results obtained after reevaluating data from our previous study [18] using the currently accepted species and the recently proposed species demarcation criterion by the ICTV. ^3^ Results obtained analyzing the newly described sequences. ^4^ Genus assignment for the described sequences. ^5^ Number of new species (percentage with respect to the total number of described sequences for each genus is given between brackets). ^6^ Number of species that cluster with at least one new sequence (percentage with respect to the total number of species is given between brackets). Novel species identified from our previous study were also used as reference species on subsequent phylogenetic and pairwise identity analyses.

		Cebriá et al. (2021) ^2^			This Study ^3^		
	Species ^1^	Sequences ^4^	Novel Species ^5^	Coincident Clusters (%) ^6^	Sequences ^4^	Novel Species ^5^	Coincident Clusters (%) ^6^
**TTV**	26	68	6 (8.8)	13 (50.0)	160	6 (3.8)	20 (62.5)
**TTMV**	38	29	11 (37.9)	11 (28.9)	111	27 (24.3)	24 (49.0)
**TTMDV**	15	17	9 (52.9)	5 (33.3)	61	17 (27.9)	16 (66.6)
**Total**	79	114	26 (22.8)	29 (36.7)	332	50 (15.1)	60 (57.1)

**Table 3:** Summary of HPgV analysis. Estimates were obtained using accession U44402 as the reference sequence. * This pool consists of two different HPgV isolates, and individual estimates cannot be independently provided.

Sample/Pool	# Reads	Average Depth Coverage	Genome Coverage	Polyprotein Coverage
SP1	25,965	1010.7	98.4	98.6
SP3	3669	130.1	94.2	95.6
SP4	4250	157.7	92.5	93.8
SP11	5706	204.0	99.2	100.0
SP13	1173	40.7	96.4	97.4
SP16	9	2.4	5.5	6.0
SP19	339	12.4	82.7	89.9
SP20	373	13.5	70.2	76.4
SP29	6606	228.7	98.2	99.2
SP30	6924	230.1	89.7	91.1
SP33	2033	71.1	92.6	94.8
SP38	2261	82.7	99.6	100.0
SP44	10,713	392.0	99.1	100.0
SP49	5226	165.7	83.4	84.0
SP53 *	5344	181.2	97.5	99.8
SP56	3523	121.1	91.7	93.3
SP58	2158	73.2	93.6	94.7

## Data Availability

The raw sequence reads were deposited in the Short Read Archive of GenBank under accession number PRJNA731624. The newly described sequences belonging to anelloviruses, HPgV, and a single microvirus were deposited in GenBank under accession numbers MZ285962-MZ286225 (Supplementary Table S4), MZ420565-MZ420581 (Supplementary Table S12), and MZ286294, respectively.

## Supplementary Materials

The following are available online at https://www.mdpi.com/article/10.3390/v13112322/s1, Figure S1: Global phylogenetic tree for the ORF1 of the three anellovirus genera, Figure S2: Phylogenetic tree including reference species from TTV genus and potential novel species from our previous study, Figure S3: HPgV phylogenetic network obtained using SplitsTree4 (GTR+Г+I, Г=0.7028, I=0.5234), Table S1: Results of viral taxonomic classification using Centrifuge for controls and samples, Table S2: Summary of taxonomic classification results for the 60 pools analyzed, Table S3: Summary of the best blastp hits for the six putative ORFs found at the new microvirus sequence, Table S4: List of anellovirus sequences/contigs detected in our study with the metaSPAdes analysis, Table S5: List of anellovirus isolates downloaded from Genbank, Table S6: Pairwise nucleotide identity matrix obtained using ORF1 from TTV sequences belonging to reference species and sequences described in our previous study [18], Table S7: Pairwise nucleotide identity matrix obtained using ORF1 from TTMV sequences belonging to reference species and sequences described in our previous study [18], Table S8: Pairwise nucleotide identity matrix obtained using ORF1 from TTMDV sequences belonging to reference species and sequences described in our previous study [18], Table S9: Pairwise nucleotide identity matrix obtained using ORF1 from TTV sequences belonging to reference species, potential novel species from our previous study, and sequences described in the present study, Table S10: Pairwise nucleotide identity matrix obtained using ORF1 from TTMV sequences belonging to reference species, potential novel species from our previous study, and sequences described in the present study, Table S11: Pairwise nucleotide identity matrix obtained using ORF1 from TTMDV sequences belonging to reference species, potential novel species from our previous study, and sequences described in the present study, Table S12: List of HPgV isolates downloaded from Genbank and described in the present study, Table S13: Demographic information of plasma donors included in each pool.

## References

[1] Holmes E.C. (2011). What does virus evolution tell us about virus origins?. J. Virol..

[2] Holmes E.C., Rambaut A., Andersen K.G. (2018). Pandemics: Spend on surveillance, not prediction. Nature.

[3] Zhang Y.-Z., Shi M., Holmes E.C. (2018). Using Metagenomics to Characterize an Expanding Virosphere. Cell.

[4] French R.K., Holmes E.C. (2020). An Ecosystems Perspective on Virus Evolution and Emergence. Trends Microbiol..

[5] Roossinck M.J. (2015). Plants, viruses and the environment: Ecology and mutualism. Virology.

[6] Kernbauer E., Ding Y., Cadwell K. (2014). An enteric virus can replace the beneficial function of commensal bacteria. Nature.

[7] Furuta R.A., Sakamoto H., Kuroishi A., Yasiui K., Matsukura H., Hirayama F. (2015). Metagenomic profiling of the viromes of plasma collected from blood donors with elevated serum alanine aminotransferase levels. Transfusion.

[8] Law J., Jovel J., Patterson J., Ford G., O’keefe S., Wang W., Meng B., Song D., Zhang Y., Tian Z. (2013). Identification of Hepatotropic Viruses from Plasma Using Deep Sequencing: A Next Generation Diagnostic Tool. PLoS ONE.

[9] Popgeorgiev N., Boyer M., Fancello L., Monteil S., Robert C., Rivet R., Nappez C., Azza S., Chiaroni J., Raoult D. (2013). Marseillevirus-like virus recovered from blood donated by asymptomatic humans. J. Infect. Dis..

[10] Stremlau M.H., Andersen K.G., Folarin O.A., Grove J.N., Odia I., Ehiane P.E., Omoniwa O., Omoregie O., Jiang P.P., Yozwiak N.L. (2015). Discovery of Novel Rhabdoviruses in the Blood of Healthy Individuals from West Africa. PLoS Negl. Trop. Dis..

[11] Kapoor A., Kumar A., Simmonds P., Bhuva N., Chauhan L.S., Lee B., Sall A.A., Jin Z., Morse S.S., Shaz B. (2015). Virome analysis of transfusion recipients reveals a novel human virus that shares genomic features with hepaciviruses and pegiviruses. MBio.

[12] Spandole S., Berca L.M., Miha G. (2015). Human anelloviruses: An update of molecular, epidemiological and clinical aspects. Arch. Virol..

[13] Stapleton J.T., Foung S., Muerhoff A.S., Bukh J., Simmonds P. (2011). The GB viruses: A review and proposed classification of GBV-A, GBV-C (HGV), and GBV-D in genus Pegivirus within the family Flaviviridae. J. Gen. Virol..

[14] Chivero E.T., Stapleton J.T. (2015). Tropism of human pegivirus (Formerly known as GB virus C/hepatitis G virus) and host immunomodulation: Insights into a highly successful viral infection. J. Gen. Virol..

[15] Kaczorowska J., Hoek L. (2020). Van Der. Human anelloviruses: Diverse, omnipresent and commensal members of the virome. FEMS Microbiol. Rev..

[16] Bhattarai N., Stapleton J.T. (2012). GB virus C: The good boy virus?. Trends Microbiol..

[17] Ataei B., Emami Naeini A., Khorvash F., Yazdani M.R., Javadi A.-A. (2012). Prevalence of transfusion transmitted virus infection in hemodialysis patients and injection drug users compared to healthy blood donors in Isfahan, Iran. Gastroenterol. Res. Pract..

[18] Cebriá-Mendoza M., Arbona C., Larrea L., Díaz W., Arnau V., Peña C., Bou J.V., Sanjuán R., Cuevas J.M. (2021). Deep viral blood metagenomics reveals extensive anellovirus diversity in healthy humans. Sci. Rep..

[19] Muhire B.M., Varsani A., Martin D.P. (2014). SDT: A Virus Classification Tool Based on Pairwise Sequence Alignment and Identity Calculation. PLoS ONE.

[20] Virgin H.W., Wherry E.J., Ahmed R. (2009). Redefining Chronic Viral Infection. Cell.

[21] Tyschik E.A., Rasskazova A.S., Degtyareva A.V., Rebrikov D.V., Sukhikh G.T. (2018). Torque teno virus dynamics during the first year of life. Virol. J..

[22] Mohr E.L., Stapleton J.T. (2009). GB virus type C interactions with HIV: The role of envelope glycoproteins. J. Viral Hepat..

[23] Feng Y., Zhao W., Feng Y., Dai J., Li Z., Zhang X., Liu L., Bai J., Zhang H., Lu L. (2011). A novel genotype of GB virus C: Its identification and predominance among injecting drug users in Yunnan, China. PLoS ONE.

[24] Ghai R.R., Sibley S.D., Lauck M., Dinis J.M., Bailey A.L., Chapman C.A., Omeja P., Friedrich T.C., O’Connor D.H., Goldberg T.L. (2013). Deep sequencing identifies two genotypes and high viral genetic diversity of human pegivirus (GB virus C) in rural Ugandan patients. J. Gen. Virol..

[25] Lauck M., Bailey A.L., Andersen K.G., Goldberg T.L., Sabeti P.C., O’Connor D.H. (2015). GB virus C coinfections in west African Ebola patients. J. Virol..

[26] Sauvage V., Eloit M. (2016). Viral metagenomics and blood safety. Transfus. Clin. Biol..

[27] Ewels P., Magnusson M., Lundin S., Käller M. (2016). MultiQC: Summarize analysis results for multiple tools and samples in a single report. Bioinformatics.

[28] Bushnell B., Rood J., Singer E. (2017). BBMerge—Accurate paired shotgun read merging via overlap. PLoS ONE.

[29] Kim D., Song L., Breitwieser F.P., Salzberg S.L. (2016). Centrifuge: Rapid and sensitive classification of metagenomic sequences. Genome Res..

[30] Martí J.M. (2019). Recentrifuge: Robust comparative analysis and contamination removal for metagenomics. PLoS Comput. Biol..

[31] Nurk S., Meleshko D., Korobeynikov A., Pevzner P.A. (2017). metaSPAdes: A new versatile metagenomic assembler. Genome Res..

[32] Edgar R.C. (2004). MUSCLE: Multiple sequence alignment with high accuracy and high throughput. Nucleic Acids Res..

[33] Kumar S., Stecher G., Li M., Knyaz C., Tamura K. (2018). MEGA X: Molecular Evolutionary Genetics Analysis across Computing Platforms. Mol. Biol. Evol..

[34] Subramanian B., Gao S., Lercher M.J., Hu S., Chen W. (2019). Evolview v3: A webserver for visualization, annotation, and management of phylogenetic trees. Nucleic Acids Res..

[35] Huson D.H., Bryant D. (2006). Application of phylogenetic networks in evolutionary studies. Mol. Biol. Evol..

[36] Shoaib M., Baconnais S., Mechold U., Le Cam E., Lipinski M., Ogryzko V. (2008). Multiple displacement amplification for complex mixtures of DNA fragments. BMC Genom..

[37] Asplund M., Kjartansdóttir K.R., Mollerup S., Vinner L., Fridholm H., Herrera J.A.R., Friis-Nielsen J., Hansen T.A., Jensen R.H., Nielsen I.B. (2019). Contaminating viral sequences in high-throughput sequencing viromics: A linkage study of 700 sequencing libraries. Clin. Microbiol. Infect..

[38] Phan T.G., Desnues C., Switzer W.M., Djoko C.F., Schneider B.S., Deng X., Delwart E. (2015). Absence of giant blood Marseille-like virus DNA detection by polymerase chain reaction in plasma from healthy US blood donors and serum from multiply transfused patients from Cameroon. Transfusion.

[39] Sauvage V., Livartowski A., Boizeau L., Servant-Delmas A., Lionnet F., Lefrère J.-J., Laperche S. (2014). No evidence of Marseillevirus-like virus presence in blood donors and recipients of multiple blood transfusions. J. Infect. Dis..

[40] De Souza W.M., Fumagalli M.J., De Araujo J., Sabino-Santos G., Gonçalves F., Maia M., Farignoli M., Modha S., Schiavo M., Helena L. (2018). Discovery of novel anelloviruses in small mammals expands the host range and diversity of the Anelloviridae. Virology.

[41] Ninomiya M., Nishizawa T., Takahashi M., Lorenzo F.R., Shimosegawa T., Okamoto H. (2007). Identification and genomic characterization of a novel human torque teno virus of 3.2 kb. J. Gen. Virol..

[42] Hsiao K., Wang L., Lin C., Liu H. (2016). New Phylogenetic Groups of Torque Teno Virus Identified in Eastern Taiwan Indigenes. PLoS ONE.

[43] De Vlaminck I., Khush K.K., Strehl C., Kohli B., Luikart H., Neff N.F., Okamoto J., Snyder T.M., Cornfield D.N., Nicolls M.R. (2013). Temporal Response of the Human Virome to Immunosuppression and Antiviral Therapy. Cell.

[44] Ninomiya M., Takahashi M., Hoshino Y., Ichiyama K., Simmonds P., Okamoto H. (2009). Analysis of the entire genomes of torque teno midi virus variants in chimpanzees: Infrequent cross-species infection between humans and chimpanzees. J. Gen. Virol..

[45] Forns X., Fernández-Llama P., Costa J., López-Labrador F.X., Ampurdanés S., Olmedo E., Saiz J.C., Guilera M., López-Pedret J., Sánchez-Tapias J.M. (1997). Hepatitis G virus infection in a haemodialysis unit: Prevalence and clinical implications. Nephrol. Dial. Transplant..

[46] Martin D.P., Murrell B., Golden M., Khoosal A., Muhire B. (2015). RDP4: Detection and analysis of recombination patterns in virus genomes. Virus Evol..

[47] Shi M., Zhang Y.-Z., Holmes E.C. (2018). Meta-transcriptomics and the evolutionary biology of RNA viruses. Virus Res..

[48] Drexler J.F., Corman V.M., Müller M.A., Lukashev A.N., Gmyl A., Coutard B., Adam A., Ritz D., Leijten L.M., van Riel D. (2013). Evidence for Novel Hepaciviruses in Rodents. PLOS Pathog..

[49] Arze C.A., Springer S., Dudas G., Patel S., Bhattacharyya A., Swaminathan H., Brugnara C., Delagrave S., Ong T., Kahvejian A. (2021). Global genome analysis reveals a vast and dynamic anellovirus landscape within the human virome. Cell Host Microbe.

[50] Thom K., Morrison C., Lewis J.C.M., Simmonds P. (2003). Distribution of TT virus (TTV), TTV-like minivirus, and related viruses in humans and nonhuman primates. Virology.

[51] Okamoto H., Takahashi M., Nishizawa T., Tawara A., Fukai K., Muramatsu U., Naito Y., Yoshikawa A. (2002). Genomic characterization of TT viruses (TTVs) in pigs, cats and dogs and their relatedness with species-specific TTVs in primates and tupaias. J. Gen. Virol..

[52] Reshetnyak V.I., Maev I.V., Burmistrov A.I., Chekmazov I.A., Karlovich T.I. (2020). Torque teno virus in liver diseases: On the way towards unity of view. World J. Gastroenterol..

[53] Spandole-Dinu S., Cimponeriu D.G., Crăciun A.-M., Radu I., Nica S., Toma M., Alexiu O.A., Iorga C.S., Berca L., Nica R. (2018). Prevalence of human anelloviruses in Romanian healthy subjects and patients with common pathologies. BMC Infect. Dis..

[54] Strassl R., Schiemann M., Doberer K., Görzer I., Puchhammer-Stöckl E., Eskandary F., Kikić Ž., Gualdoni G.A., Vossen M.G., Rasoul-Rockenschaub S. (2018). Quantification of Torque Teno Virus Viremia as a Prospective Biomarker for Infectious Disease in Kidney Allograft Recipients. J. Infect. Dis..

[55] Frye B.C., Bierbaum S., Falcone V., Köhler T.C., Gasplmayr M., Hettich I., Dürk T., Idzko M., Zissel G., Hengel H. (2019). Kinetics of Torque Teno Virus-DNA Plasma Load Predict Rejection in Lung Transplant Recipients. Transplantation.

[56] Chandriani S., Skewes-Cox P., Zhong W., Ganem D.E., Divers T.J., Van Blaricum A.J., Tennant B.C., Kistler A.L. (2013). Identification of a previously undescribed divergent virus from the Flaviviridae family in an outbreak of equine serum hepatitis. Proc. Natl. Acad. Sci. USA.

[57] Bonsall D., Gregory W.F., Ip C.L.C., Donfield S., Iles J., Ansari M.A., Piazza P., Trebes A., Brown A., Frater J. (2016). Evaluation of Viremia Frequencies of a Novel Human Pegivirus by Using Bioinformatic Screening and PCR. Emerg. Infect. Dis..

[58] Pavesi A. (2001). Origin and evolution of GBV-C/hepatitis G virus and relationships with ancient human migrations. J. Mol. Evol..

[59] Sharp P.M., Simmonds P. (2011). Evaluating the evidence for virus/host co-evolution. Curr. Opin. Virol..

[60] Marano G., Franchini M., Farina B., Piccinini V., Pupella S., Vaglio S., Grazzini G., Liumbruno G.M. (2017). The human pegivirus: A new name for an “ancient” virus. Can transfusion medicine come up with something new?. Acta Virol..

[61] Jordier F., Deligny M.-L., Barré R., Robert C., Galicher V., Uch R., Fournier P.-E., Raoult D., Biagini P. (2018). Human pegivirus isolates characterized by deep sequencing from hepatitis C virus-RNA and human immunodeficiency virus-RNA-positive blood donations, France. J. Med. Virol..

[62] Parreira R., Branco C., Piedade J., Esteves A. (2012). GB virus C (GBV-C) evolutionary patterns revealed by analyses of reference genomes, E2 and NS5B sequences amplified from viral strains circulating in the Lisbon area (Portugal). Infect. Genet. Evol..

[63] Worobey M., Holmes E.C. (2001). Homologous recombination in GB virus C/hepatitis G virus. Mol. Biol. Evol..

[64] Blackard J.T., Ma G., Polen C., DuBois J.C., Gast J., Radens C.M., Sterling R.K., Sherman K.E. (2016). Recombination among GB virus C (GBV-C) isolates in the United States. J. Gen. Virol..

[65] Neibecker M., Schwarze-Zander C., Rockstroh J.K., Spengler U., Blackard J.T. (2011). Evidence for extensive genotypic diversity and recombination of GB virus C (GBV-C) in Germany. J. Med. Virol..

[66] Wu H., Padhi A., Xu J., Gong X., Tien P. (2016). Evidence for within-host genetic recombination among the human pegiviral strains in HIV infected subjects. PLoS ONE.

[67] Smith D.B., Basaras M., Frost S., Haydon D., Cuceanu N., Prescott L., Kamenka C., Millband D., Sathar M.A., Simmonds P. (2000). Phylogenetic analysis of GBV-C/hepatitis G virus. J. Gen. Virol..

[68] Muerhoff A.S., Tillmann H.L., Manns M.P., Dawson G.J., Desai S.M. (2003). GB virus C genotype determination in GB virus-C/HIV co-infected individuals. J. Med. Virol..

[69] Schwarze-Zander C., Blackard J.T., Zheng H., Addo M.M., Lin W., Robbins G.K., Sherman K.E., Zdunek D., Hess G., Chung R.T. (2006). GB virus C (GBV-C) infection in hepatitis C virus (HCV)/HIV-coinfected patients receiving HCV treatment: Importance of the GBV-C genotype. J. Infect. Dis..

[70] Alcalde R., Nishiya A., Casseb J., Inocêncio L., Fonseca L.A.M., Duarte A.J.S. (2010). Prevalence and distribution of the GBV-C/HGV among HIV-1-infected patients under anti-retroviral therapy. Virus Res..

[71] Blackard J.T., Ma G., Welge J.A., Taylor L.E., Mayer K.H., Klein R.S., Celentano D.D., Sobel J.D., Jamieson D.J., King C.C. (2017). Cytokine/chemokine expression associated with Human Pegivirus (HPgV) infection in women with HIV. J. Med. Virol..

[72] Berzsenyi M.D., Bowden D.S., Roberts S.K., Revill P.A. (2009). GB virus C genotype 2 predominance in a hepatitis C virus/HIV infected population associated with reduced liver disease. J. Gastroenterol. Hepatol..

[73] Miao Z., Gao L., Song Y., Yang M., Zhang M., Lou J., Zhao Y., Wang X., Feng Y., Dong X. (2017). Prevalence and Clinical Impact of Human Pegivirus-1 Infection in HIV-1-Infected Individuals in Yunnan, China. Viruses.

[74] Greenhalgh S., Schmidt R., Day T. (2019). Fighting the Public Health Burden of AIDS With the Human Pegivirus. Am. J. Epidemiol..

[75] Maidana-Giret M.T., Silva T.M., Sauer M.M., Tomiyama H., Levi J.E., Bassichetto K.C., Nishiya A., Diaz R.S., Sabino E.C., Palacios R. (2009). GB virus type C infection modulates T-cell activation independently of HIV-1 viral load. AIDS.

[76] Bhattarai N., Rydze R.T., Chivero E.T., Stapleton J.T. (2012). GB virus C viremia is associated with higher levels of double-negative T cells and lower T-cell activation in HIV-infected individuals receiving antiretroviral therapy. J. Infect. Dis..

[77] Chang C.M., Stapleton J.T., Klinzman D., McLinden J.H., Purdue M.P., Katki H.A., Engels E.A. (2014). GBV-C infection and risk of NHL among U.S. adults. Cancer Res..

[78] Krajden M., Yu A., Braybrook H., Lai A.S., Mak A., Chow R., Cook D., Tellier R., Petric M., Gascoyne R.D. (2010). GBV-C/hepatitis G virus infection and non-Hodgkin lymphoma: A case control study. Int. J. Cancer.

[79] Kapoor A., Simmonds P., Scheel T.K.H., Hjelle B., Cullen J.M., Burbelo P.D., Chauhan L.V., Duraisamy R., Sanchez Leon M., Jain K. (2013). Identification of rodent homologs of hepatitis C virus and pegiviruses. MBio.

[80] Sibley S.D., Lauck M., Bailey A.L., Hyeroba D., Tumukunde A., Weny G., Chapman C.A., O’Connor D.H., Goldberg T.L., Friedrich T.C. (2014). Discovery and characterization of distinct simian pegiviruses in three wild African Old World monkey species. PLoS ONE.

