# Supplementary material for: Exploring the Diversity of the Human Blood Virome
Source: Viruses. 2021 Nov 21;13(11):2322. doi: 10.3390/v13112322 (PMC8621239; doi:10.3390/v13112322)

Supplementary Figure S1. Global phylogenetic tree for the ORF1 of the three anellovirus genera. Downloaded sequences defined as reference species and sequences described in this study (labelled in red) are included. 0.7-0.85 and 0.85-1.0 bootstrap value ranges are indicated with blue and red circles, respectively. Scale bar indicates the evolutionary distance in nucleotide substitutions per site.

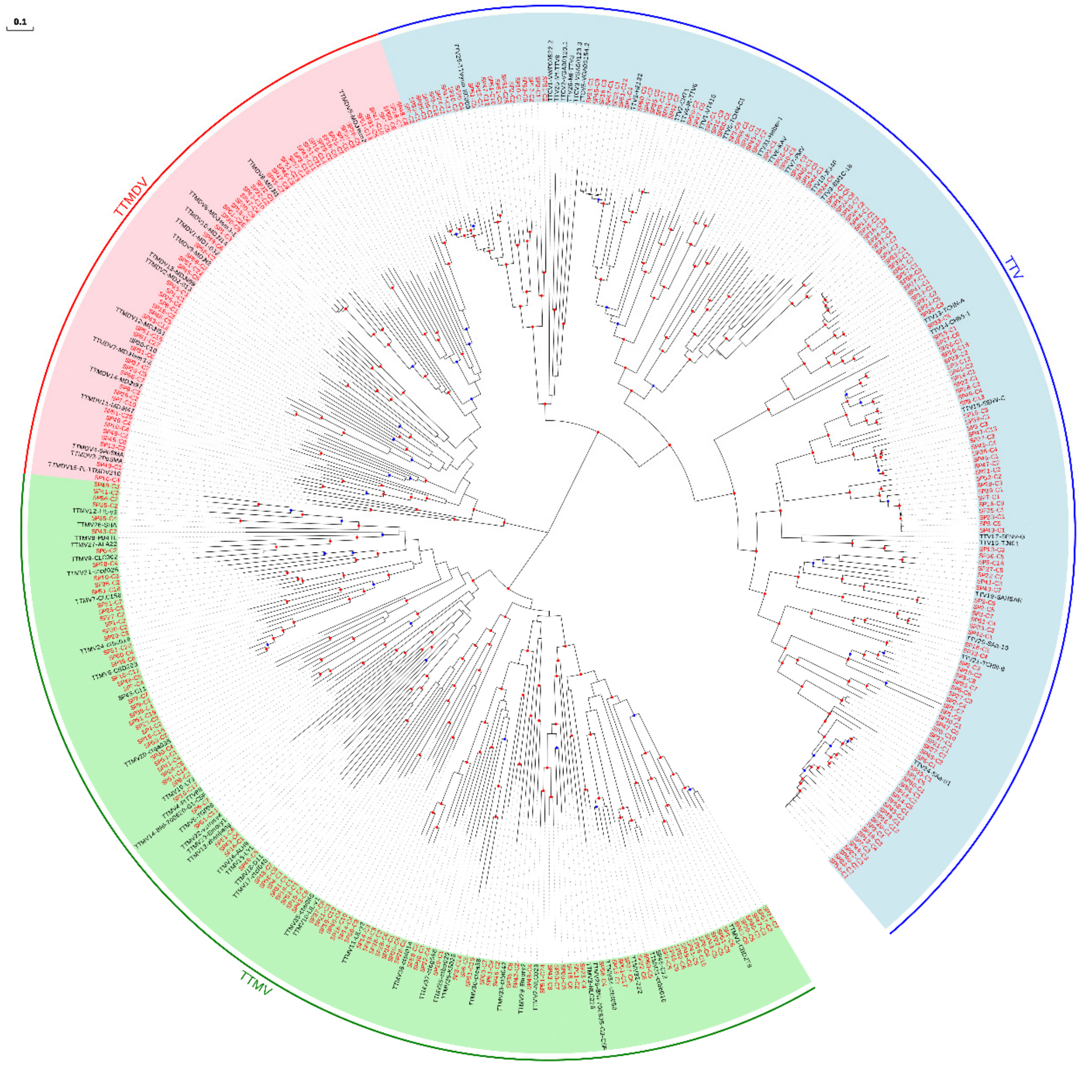

Supplement: Supplementary file 1 [file viruses-13-02322-s001.zip › Supplementary Figure S1.pdf]
