# Supplementary material for: Exploring the Diversity of the Human Blood Virome
Source: Viruses. 2021 Nov 21;13(11):2322. doi: 10.3390/v13112322 (PMC8621239; doi:10.3390/v13112322)

Supplementary Figure S2. Phylogenetic tree including reference species from TTV genus and potential novel species from our previous study. Sequences described in this study that can be considered as new species are also included and labelled in red. Numbers between brackets indicate the number of new sequences clustering with a specific genotype (All new sequences are explicitly shown in Fig. 2). Bootstrap values are indicated in red at each branch point. Scale bar indicates the evolutionary distance in nucleotide substitutions per site.

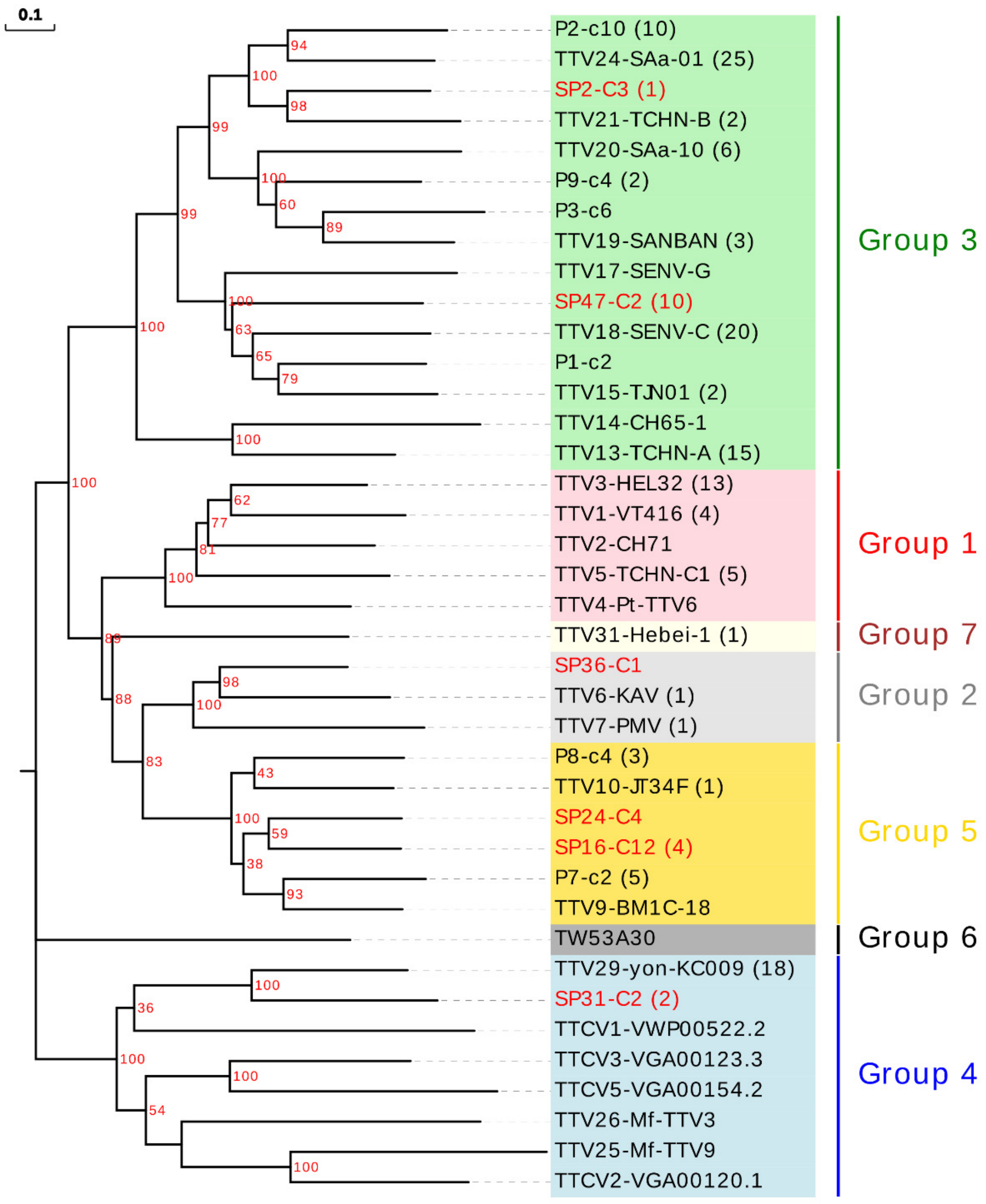

Supplement: Supplementary file 1 [file viruses-13-02322-s001.zip › Supplementary Figure S2.pdf]
