# Supplementary figures and images for: Exploring the Diversity of the Human Blood Virome
Source: Viruses. 2021 Nov 21;13(11):2322. doi: 10.3390/v13112322 (PMC8621239; doi:10.3390/v13112322)

## Slide 1
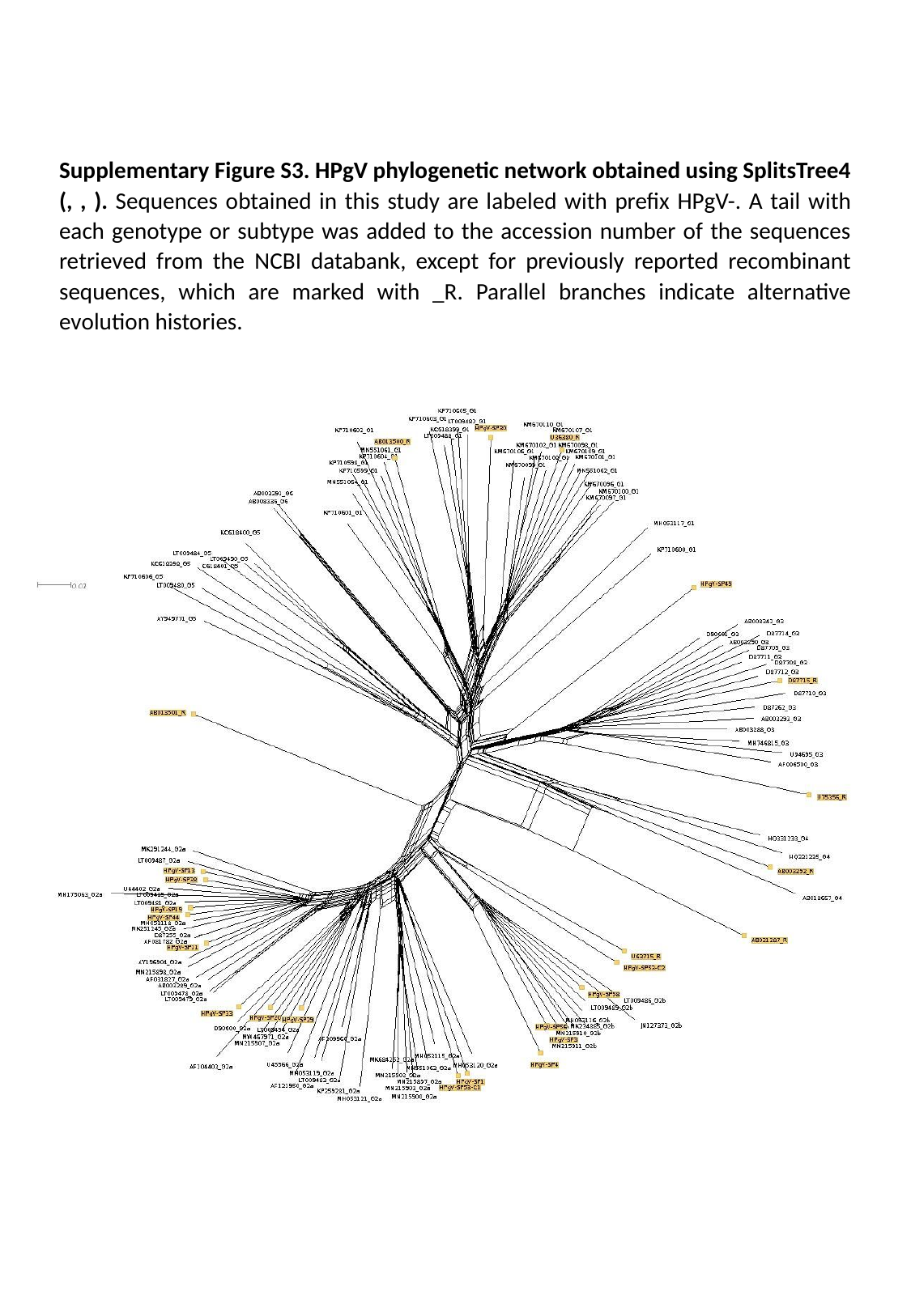

Supplement: Supplementary file 1 [file viruses-13-02322-s001.zip › Supplementary Figure S3.pptx]
